# Practical nutritional recovery strategies for elite soccer players when limited time separates repeated matches

**DOI:** 10.1186/s12970-017-0193-8

**Published:** 2017-09-12

**Authors:** Mayur Krachna Ranchordas, Joel T. Dawson, Mark Russell

**Affiliations:** 1 Sheffield Hallam University, Academy of Sport & Physical Activity, A220 Collegiate Hall, Collegiate Crescent Campus, Sheffield, S102BP UK; 2 Stoke City Football Club, bet365 Stadium, Stanley Matthews Way, Stoke-on-Trent, ST4 4EG UK; 3 School of Social and Health Sciences, Leeds Trinity University, Horsforth, Leeds, LS18 5HD UK

**Keywords:** Soccer, Nutrition, Recovery, Polyphenols, Omega-3, Creatine, Fixture, Congestion

## Abstract

Specific guidelines that aim to facilitate the recovery of soccer players from the demands of training and a congested fixture schedule are lacking; especially in relation to evidence-based nutritional recommendations. The importance of repeated high level performance and injury avoidance while addressing the challenges of fixture scheduling, travel to away venues, and training commitments requires a strategic and practically feasible method of implementing specific nutritional strategies. Here we present evidence-based guidelines regarding nutritional recovery strategies within the context of soccer. An emphasis is placed on providing practically applicable guidelines for facilitation of recovery when multiple matches are played within a short period of time (i.e. 48 h). Following match-play, the restoration of liver and muscle glycogen stores (via consumption of ~1.2 g⋅kg^−1^⋅h^−1^ of carbohydrate) and augmentation of protein synthesis (via ~40 g of protein) should be prioritised in the first 20 min of recovery. Daily intakes of 6–10 g⋅kg^−1^ body mass of carbohydrate are recommended when limited time separates repeated matches while daily protein intakes of >1.5 g⋅kg^−1^ body mass should be targeted; possibly in the form of multiple smaller feedings (e.g., 6 × 20–40 g). At least 150% of the body mass lost during exercise should be consumed within 1 h and electrolytes added such that fluid losses are ameliorated. Strategic use of protein, leucine, creatine, polyphenols and omega-3 supplements could also offer practical means of enhancing post-match recovery.

## Background

**Fig. 1:**
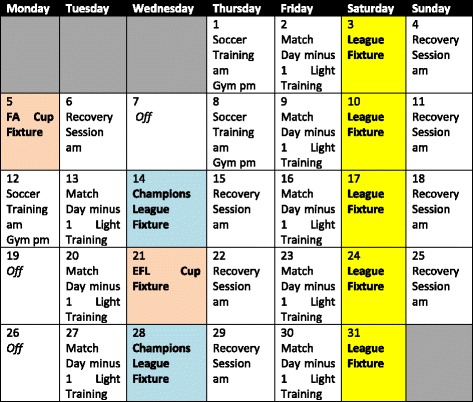
A Typical monthly schedule for a top professional soccer club in the Premier League

Over the course of a 45 week season, professional European soccer teams may play in excess of 60 competitive matches [1, 2] and thus at specific times of the year, multiple matches will be played within a single week [1]. Notwithstanding the additional match demands of the pre-season period, it is common for players to compete in 2–3 matches within an 8 day period (see Fig. 1 for a typical weekly schedule for an English Premier League team) on multiple occasions throughout the season. It should be noted that the notion of limited recovery between soccer matches is not unique to the English Premier League as fixture congestion is also common among U.S. University teams as well as youth teams who play multiple games in a weekend. Up to 120 h are required to restore disturbances in metabolic and physical performance indices that result from soccer match-play [3]. Injury risk has been observed to increase when less than 96 h separates games [1, 2] and the reduced recovery time between matches played in FIFA World Cup competitions is perceived to be a primary cause of injury in professional soccer players [4]. Therefore, the ability to facilitate post-match recovery is desirable.

Accumulated fatigue can arise from a repetition of matches and training performed within a short period of time (e.g., daily training with matches separated by ~48 h; 5). The demands of a congested fixture schedule means that recovery duration may sometimes be less than optimal when seeking to maintain physical performance and a low injury rate. Indeed, a reported 6.2-fold higher injury rate occurred in players who played two matches a week compared to one and the majority of these injuries (i.e., 76%) were reported to be caused by overuse [5]. As muscle injuries constitute almost one third of the time lost in men’s professional soccer [6], it appears that amassed fatigue during a congested fixture period may contribute to both underperformance and/or elevated injury risk [3]; particularly in the final 15 min of a match [7]. Notably, when three games are played within a week, repeated sprint performance is compromised (more so after the second game) and muscle soreness is increased, knee range of motion is impaired, and muscle damage, oxidative stress, and inflammatory markers are perturbed [8]. While it must be acknowledged that the extent of exposure to periods of match congestion in professional soccer players may be limited [9], when such periods do occur, performance is likely compromised and injury risk may be elevated [7].

A number of interventions have been proposed to facilitate post-exercise recovery, including, but not limited to: cold water immersion, active recovery, compression garments, massage and electrical stimulation [10]; many of which are routinely used by professional soccer teams. However, nutritional strategies are amongst the most popular and accessible methods of facilitating restoration of performance and physiological perturbations following soccer-specific exercise. Despite the popularity of soccer, surprisingly few guidelines exist that seek to address the practical application of nutrition for recovery from soccer when limited time (e.g., ~48 h) separates matches. The importance of optimised recovery strategies is particularly prevalent when congested fixture periods exist and implementation of them may be complicated by logistical issues such as late fixture times and demanding travel schedules. Therefore, the purpose of this review was to evaluate current knowledge regarding nutritional recovery strategies within the context of soccer. Our emphasis is on providing contextually relevant recommendations for facilitation of post-exercise recovery when multiple matches are played within a short period of time. We present practical strategies relating to the composition, quantity and timing of nutritional intake for the elite soccer player wishing to improve their recovery via evidence-based dietary strategies. Practical issues concerning the implementation of such strategies within the elite environment are also considered.

## Method

Articles were retrieved in accordance with an extensive search in several databases including MEDLINE (1966–2016); SPORTDiscus (1966–2016); PubMed (1966–2016) and Google Scholar (1980–2015). The following search terms were used in various combinations: “recovery,” “nutrition,” “diet,” “food,” “soccer,” “football,” “supplements,” “ergogenic aids,” “glycogen re-synthesis,” “refuelling,” “repair,” “adaptation.” Only studies that were conducted using human participants were selected and references cited in the retrieved articles were also considered for inclusion.

### Characterising the demands of soccer match-play

Soccer is a physically demanding intermittent sport which consists of recurrent high-intensity running, intensive soccer-specific actions and requirements for a high endurance capacity [11]. The game demands an ability to intersperse repeated actions at maximal or near-maximal intensity with periods of low-to-moderate intensity (including active recovery or passive rest) [12]. Accordingly, both anaerobic and aerobic energy pathways are required during match-play [11] as players typically cover distances of 9–12 km, and perform ~1350 activities (including a change of movement every 4–6 s) while executing ~220 runs at high speed [11] over 90 min; responses which may be exacerbated by involvement in extra-time [13, 14] in tournament scenarios.

Mean energy expenditure for soccer match-play has been estimated to be ~1106 kcal [15], and between 3439 and 3822 kcal·day^−1^ for players undertaking daily training [16, 17]. Using the doubly labelled water technique, English Premier League soccer players have exhibited a state of energy balance (i.e., matching energy intake and expenditure: 3186 ± 368 vs 3566 ± 585 kcal·day^−1^, respectively; [18], yet previous data from youth soccer players and using estimated markers of energy balance do not support such findings [19, 20]. Indeed, in youth players it has been reported that a mean daily energy deficit of 310 ± 399 kcal·day^−1^ is common and heavy training days and matches result in the largest energy deficits of 502 ± 533 kcal·day^−1^ [19]. It has also been reported that carbohydrate intake on a match-day and in the time preceding match-play was less than optimal [18]. While highlighting a possible performance-enhancement strategy to a single match, the implications of such practices may be compounded when multiple matches are played with a short turn-around time as the recovery nutrition in professional players may be compromised [18].

Similarly, in players with energy intakes that fail to balance expenditure, the predisposition of injury, accentuation of fatigue and suppression of the immune system may occur [21, 22]. On a day to day basis, there are large changes in energy expenditure depending on the type, intensity and duration of training [23]. Moreover, variability exists in the activity and energy demands of players that are dependent on the individual and position played within a team; both of which can be dictated by extraneous factors such as tactical role, quality of opponent, style of playing, and environmental factors [11]. It is therefore important that players periodise energy and macronutrient intake, particularly carbohydrate, according to requirements.

The importance of carbohydrate for soccer has been acknowledged since the early 1970’s when muscle biopsy techniques identified compromised muscle glycogen stores following soccer match-play; a finding which had negative ramifications for performance [24]. Indeed, a better between-half maintenance of total distance covered and higher movement intensities were achieved by players starting the game with higher muscle glycogen concentrations [24]. More recent studies have demonstrated a fibre-specific reduction in muscle glycogen concentration [25] with knee extensor maximal voluntary activation and peak torque responses shown to also be reduced [26]. Accordingly, over the course of 90 min, the intensity and frequency of explosive actions tend to reduce, resulting in a transient decline in physical performance [3]. Likewise, a high degree of muscle damage occurs as a result of exhaustive intermittent activities and regular unexpected changes of direction [27]. Consequently, refuelling and recovery nutrition are crucial components to promote muscle recovery and glycogen resynthesis. Additionally, recovery modalities and the nutrient intake/timing approach need to be strategically integrated to fully maximise muscle recovery and soccer-specific adaptations.

### Recovery nutrition strategies

A clearly planned nutritional strategy can likely assist practitioners to facilitate the replenishing of glycogen stores, acceleration of muscle-damage repair and enhanced rehydration; all of which seek to improve subsequent performance. Commencing the immediate recovery phase as close to the end of the match as is reasonably possible will likely confer beneficial effects before continuation for several hours after until sleep occurs.

#### Refuelling after a match – The immediate recovery phase

The main focus immediately after a match is to replenish both liver and muscle glycogen stores through ingestion of adequate carbohydrate. For optimum glycogen resynthesis it is a prudent strategy to consume carbohydrate immediately after a game as glycogen-synthesising enzymes are most active during this time [28]; thus there is a potential ‘window of opportunity’ that players should seek to take advantage of. Indeed, when compared to immediate carbohydrate ingestion, delaying carbohydrate feeding until 2 h after exercise can result in lower muscle glycogen concentrations by 45% when assessed 4 h post-exercise [28]. Thus players should be encouraged to consume a recovery drink and/or snacks as soon as possible after a match ends. This can be achieved practically by providing several opportunities to consume carbohydrate-electrolyte drinks on the pitch, in the media suites for post-match interviews and in the changing rooms.

The amount and frequency of carbohydrate ingested is an important factor to consider during the immediate recovery period (i.e., within 20 min of match-play). Generally, the ingestion of 1–1.5 g·kg^−1^·h^−1^ of carbohydrate has been shown to benefit maximal glycogen resynthesis in the first 4 h post-exercise [29]. Therefore, based on the upper limit of this recommendation, an 80 kg player would be advised to consume ~96 g of carbohydrate per hour in the hours after a game finishes, with a particular emphasis on achieving such rates during times of fixture congestion. Furthermore, during this initial stage of recovery, a strategy of frequent ingestion of carbohydrate (i.e., every 30 min) has been shown to induce greater glycogen resynthesis rates compared to a less regular (i.e., every 2 h) protocol [30]. Similarly, adding 0.2–0.5 g⋅kg^−1^⋅day^−1^ of protein to carbohydrate has been shown to stimulate glycogen resynthesis to a greater extent than consuming carbohydrate alone [31] but only when carbohydrate intake is less than 1.2 g⋅kg^−1^⋅day^−1^. It has been suggested that high glycaemic index (GI) foods may be preferable over moderate and low GI foods when the goal is to restore glycogen as quickly as possible [32–34].

The consumption of adequate quantities of carbohydrate in this post-match phase is likely the most beneficial aspect of carbohydrate recommendations. Accordingly, support staff should seek to provide food and drinks that are both tempting and practical to eat (see Tables 1, 2 and 3 for practical examples). Food options should be promoting a desire to eat such that sufficient amounts in agreement with recommended values are realised as a loss of appetite may exist in some players in the time shortly after matches. Support staff should ascertain the types of foods players are likely to eat in this immediate recovery phase as players may have individual cultural preferences.

**Table 1:** Refuelling for the Immediate Recovery Phase 0–4 h

Strategy	Food Choices
• Start to consume carbohydrate as soon as possible after the cessation of exercise taking full advantage of a ‘window of opportunity’ where high rates of glycogen storage present in the muscle. • Aim to ingest a recovery snack or meal that provides approximately 1 g·kg^−1^ body mass (e.g. 80 g for 80 kg player) per hour during the first 4 h of recovery until normal eating patterns are resumed. • This strategy should be implemented after a high intensity fuel-depleting session or game when muscle fuel stores need to be fully maximised in a short time period before the next demanding exercise bout. • Provide food and drinks that are both tempting and practical to eat that are appetite promoting so that the player will consume sufficient amounts to meet their fuel targets. The food provided could vary according to the environment in which the game is played as well as the time of the day. Support staff should ascertain the types of foods players are likely to eat in this immediate recovery phase as players may have individual cultural preferences. • Creatine ingestion with carbohydrate will help restore important phosphocreatine stores in this short period.	Recovery snacks containing 50 g of CHO: - 250–350 ml of milk-shake or fruit smoothie - 2 slices toast/bread/bagel with jam, banana or honey topping - 2 cereal bars - Large (300 g) baked potato with filling - 2 sport gels - 700-800 ml of sports drink - Fruit salad with 200 g of yoghurt - Sandwich with meat filling - Sports bar (check the label for content) - Rice cakes - Tortilla wraps with filling - Medium bowl of baked sweet potato wedges - A medium bag of popcorn - Thin base pizza slices (i.e., tortilla) with mixed toppings - Panini’s with mixed fillings

**Table 2:** Repair and Adaptation for the Immediate Recovery Phase 0–4 h

Strategy	Food Choices
• Ingest a protein-rich high quality source that provides 30–40 g of protein (containing 6–9 g of essential amino acids) as soon as possible after exercise. Leucine in particular is an important amino acid for its anabolic stimulating properties. • Plan a feeding pattern which includes this optimal protein serving of 20–25 g along with other nutritional goals every 3–5 h to fully maximise recovery in the immediate phase. • Have a protein-rich snack before bed, which preferably contains casein (e.g. 200 g of cottage cheese or 40 g in a liquid supplement) to optimise protein synthesis overnight.	Rapidly digested protein sources containing 10 g to have in the immediate recovery phase: - 300 ml milk, milkshake, flavoured milk - 20–30 g high protein sports bar (quantities dependent on the brand) - 10–15 g whey-based protein powder (quantities dependent on the brand) - 200 g Greek-style yogurt - 250 ml of low-fat custard

**Table 3:** Practical nutritional recovery strategies for elite soccer players when limited time separates repeated matches

Phase	Rationale	Practical application
Refuelling (post match) / Pre-Loading (pre match)	A player should aim to consume approximately 6–10 g·kg^−1^ of body mass (e.g. 480–800 g for an 80 kg player) of carbohydrates on the days where both muscle recovery/loading is needed (24–72 h between games). This should be coupled with a reduction in training volume/intensity. This is to be achieved through 3–4 main meals and regular carbohydrate snacking spaced out throughout the day. Fuel intake should match the demands of energy expended. Players who have been an unused sub or only played part of a game do not require the same level of energy intake as players who played the whole game. Taking in more energy than required could lead to weight gain.	• Carbohydrate sources to include as part of a nutritious meal: • Grains (quinoa, pasta, rice, noodles and couscous) • Starchy vegetables (potatoes), Legumes (beans and lentils), Fruits • Cereals (porridge, muesli) • Label foods appropriately to nudge players to increase carbohydrate portion for both match day −1 as well as post-match • Convenient food such as sweet potato wedges, chicken coated in breadcrumbs, and chicken burritos served post-match can increase uptake due to convenience
Maintenance of Repair and Adaptation Daily intake post match before subsequent fixture	During intensified periods of competition a recommended strategy of 1.5 g·kg^−1^ -2 g·kg^−1^body weight per day (e.g. 120–160 g for 80 kg player) should be sufficient to fully repair damaged muscle and stimulate soccer specific adaptation. Meals and snacks should be divided into 6 × 20–25 g protein servings over the day, interspersed by roughly 3 h to fully maximise protein synthesis rates in the days between competition.	Protein sources containing 10 g protein (add to carbohydrate sources for high quality recovery meals): • 40 g of cooked chicken, lean beef, lamb or pork. • 300 ml milk • 2 small eggs • 30 g of reduced fat cheese • 120 g tofu or soy meat • 50 g canned tuna or salmon or grilled fish
Rehydration Immediate Recovery	Rehydration should occur as soon after exercise finishes. A player should aim to intake a volume that is approximately 150–200% of the estimated deficit to account of ongoing losses (e.g. urine output) with a rough guide of 1 kg weight lost = 1.5 l of fluid required. They should aim to replace the volume lost within 2–4 h post exercise over regular time period to prevent the gastrointestinal distress associated with large fluid intakes. Key electrolytes need to be replaced – principally sodium – and this can be achieved either through electrolyte containing drinks or consuming fluids with ‘salty’ foods. Excessive alcohol consumption must be avoided as it is counterproductive to overall recovery goals.	Ultimately fluid choices need to be palatable, suit the other recovery needs of the player, practiced and are practical within their recovery environment: • Sports drinks containing electrolytes and carbohydrate • Milk based drinks/supplements which include other nutrients • Fruit juices • Cola drinks, tea and coffee could provide a valuable source of fluid and should not be totally avoided • Only have water if salty snacks are consumed at the same time
Reduce inflammation and muscle soreness Immediate Recovery	During intensified fixture congestion antioxidants and anti-inflammatory food components or supplements can modulate the inflammatory reaction may prove beneficial in the acute recovery phase. Concentrated tart cherry juice and omega-3 fish oil supplements are two supplements which may have accelerate recovery time but further research is warranted in elite team sports. It is important to note that any form of antioxidant or anti-inflammatory supplement should be carefully dosed. Soccer-specific adaptations are triggered by the inflammatory and redox reactions occurring after a strenuous exercise stimulus.	Dietary sources of antioxidants include the majority of fruits and vegetables. High antioxidant containing foods for example: • Blueberries, Prunes, Blueberries, Sprouts, Broccoli, Raspberry, Sweet cherry Dietary sources contain omega −3: • Oily fish, beans, Flax seeds, Walnuts

The type of carbohydrate recommended in the immediate phase of recovery is high GI foods (see Table 1 for examples). High GI sources are proven to accelerate muscle glycogen resynthesis rates in the first 6 h of recovery compared to low GI sources, most likely due to malabsorption of low GI carbohydrate-rich foods [35]. However, the effect of high GI carbohydrate meals on subsequent soccer-specific performance still remains unclear, with no difference observed between high and low GI diets on endurance and sprint performance 24 h after 90 min of intermittent exercise [36]. It is the player’s preference that should drive the decision as to whether solid or liquid forms of carbohydrate are ingested as both appear equally effective for muscle glycogen restoration [37].

From a practical perspective, the consumption of high amounts of carbohydrate required from food sources can bring about gastrointestinal problems so it is important that players have access to a mixture of fluid and solid foods to prevent such issues [38]. There is evidence to suggest that multiple transportable carbohydrates in the form of glucose and fructose increases gastric empting and fluid delivery compared to glucose only [39, 40] thus drinks provided at the end of the match should contain multiple transportable carbohydrates. Due to the fact that liquid carbohydrate solutions can contribute to rehydration in conjunction with exogenous carbohydrate supply, carbohydrate-containing fluids may be more preferable for immediate ingestion when compared to solid foods. A selection of high GI drinks and snacks should be readily available in the changing room after a game (refer to Table 1 for a selection of recommended carbohydrate foods).

The co-ingestion of protein with carbohydrate has proven beneficial in the context of glycogen resynthesis when sub-optimal carbohydrate amounts were consumed via an augmentation of postprandial insulin secretion [41]. A similar increase in glycogen synthetic rate has been observed when 0.4 g·kg^−1^·h^−1^ of protein was added to 0.8 g·kg^−1^·h^−1^ of carbohydrate relative to ingesting 1.2 g·kg^−1^·h^−1^ of carbohydrate alone [30]. The inclusion of protein to sufficient carbohydrate intakes is advisable to aid glycogen re-synthesis and enhance muscle tissue repair [42] (see Table 1). As milk or flavoured milk naturally contains a mixture of carbohydrate and protein, it may positively influence recovery and is likely a good choice of recovery beverage for lactose-tolerant players [43, 44].

#### Refuelling after a match - daily recovery between games

During a congested week (see Fig. 1), it is important to implement a carbohydrate feeding strategy that not only replenishes endogenous fuel stores but also seeks to fully maximise muscle glycogen concentrations in preparation for the next game as the 48 h post-exercise recovery period may also coincide with the 48 h period leading into the subsequent match. Optimal performance can largely be attributed to carbohydrate availability [45]. Notably, players consuming a high carbohydrate diet (10 g·kg^−1^·day^−1^ for one week improved repeated high intensity intermittent performance compared to players on a mixed diet (5 g·kg^−1^·day^−1^carbohydrate; [46]. However, recent soccer-specific literature has failed to report an increase in glycogen concentrations above pre-match levels 48 h after a game, despite the ingestion of a high carbohydrate diet of up to 10 g·kg^−1^·day^−1^ [11, 47]. Similarly, a carbohydrate rich diet with whey protein ingestion failed to increase glycogen resynthesis when compared to a normal diet [25]. Therefore, supercompensation of muscle glycogen concentrations has yet to be reported 48 h after a game; a response which is typically seen in sports such as cycling [48]. This may be attributed to the high eccentric component involved in soccer-specific movements with resulting muscle damage impairing glycogen resynthesis during recovery [47]. Fast twitch-muscle fibres in particular, had lower glycogen content in comparison to slow twitch fibres 48 h after a high carbohydrate diet [25]. Practically this could have implications on recovery time scale for the more ‘explosive’ players in the team who have a higher composition of these fibres in the muscle but more research is warranted in this area.

While carbohydrate recovery strategies in the 48 h after a game are less clear than endurance sports, it is difficult to recommend exact guidelines for the amount for optimal recovery. Nevertheless, a general guideline of 6–10 g·kg^−1^·day^−1^ is a prudent aim for elite soccer players in the days of muscle glycogen recovery/loading. This could be achieved through 3–4 main meals and regular carbohydrate snacking interspersed throughout the day (Table 1). This nutritional approach, coupled with acutely modulating training intensity and duration, will likely increase the availability of carbohydrate in the body in a week that involves 3 games in a 7-day period.

#### Repair and adaptation after a match – The immediate recovery phase

Exercise increases both muscle protein breakdown and protein synthesis [49]. However, prolonged periods of negative protein balance may result if synthesis rates are not periodically elevated through dietary protein consumption; a scenario that the elite player should seek to avoid when fixtures are congested. The effects of a high amount of eccentric actions during match-play, as well as impacts from tackles and challenges with the opposition, results in impaired muscle function [50] that must be restored. To repair damaged muscle fibres and stimulate molecular adaptation, the post-match nutrition strategy should target the promotion of protein synthesis and attenuation of muscle breakdown. It has recently been shown that consuming 40 g of protein rather than just 20 g after exercise stimulates greater myofibrillar protein synthesis irrespective of the lean body mass of the individual [51]. Thus, the consumption of 40 g of protein as a post-match serving seems to enhance protein synthesis rates relative to smaller doses examined previously [52, 53].

Ultimately, protein-requirements should be achieved through high quality protein meals and snacks in the diet (see Table 2). However, appetite can sometimes be suppressed following high intensity exercise so liquid supplements can be provided as an alternative for players who cannot eat solid foods. In this respect, whey protein has proven to be a superior source in comparison to soy or casein when taken in isocaloric amounts [54]. This is due to its quicker digestive properties and rapid absorption kinetics. It also contains a high proportion of the key amino acid leucine, which is believed to be the main trigger for muscle protein synthesis augmentation [55]. Animal proteins such as chicken, beef and fish can also contain a high amount of this key amino acid.

Using protein supplements can be a convenient strategy for many athletes. As previously discussed, whey protein is superior to soy and casein sources because of its rapid digestion and higher leucine content [54]. That said, plasma aminoacidemia is higher following the ingestion of liquid versus solid protein sources [56]; therefore, post-game benefits of fluid-based protein ingestion may be realised. A ready to drink formulation may also have a greater practical appeal to players post-game.

Leucine is an essential amino acid which through the activation of mammalian target of rapamycin complex (mTOR) signalling pathway may in part attenuate the decrease in muscle protein synthesis after exercise [57]. It is present in high quality proteins and it has been reported that 3 g of leucine is capable of enhancing muscle resistance to insulin through muscle protein synthesis activation [58]. This amount can be obtained through dietary sources such as 140 g of chicken, 170 g of fish or 20–25 g of whey protein, but it can also be ingested as an isolated supplement.

#### Repair and adaptation after a match - daily recovery between games

After the initial intake of protein in the hours after a game, it is important for the player to continue maximising their protein synthesis over subsequent days to support recovery and adaptation. Players should be strongly encouraged to include sources of protein in their meals with the amount of protein required daily being dependent on the severity of the player’s physical programme. Although a sedentary male is recommended to consume 0.8–1.2 g·kg^−1^·day^−1^ of protein to achieve nitrogen balance, elite soccer players will require more to support their intensified workload during busy periods. For example, a daily protein intake in the range of 2.3 g·kg^−1^·day^−1^ body mass (BM) has shown to better maintain muscle mass when there is an energy deficit [59]. Furthermore, when protein intake was elevated from 1.5 g·kg^−1^·day^−1^ to 3 g·kg^−1^·day^−1^ immune function was better preserved, resulting in less upper respiratory tract infections and an overall tolerance of strenuous training [60].

Although there is an absence in research relating to daily protein intake for elite players during intensified periods, it would be prudent to recommend that at least 1.5–2 g·kg^−1^·day^−1^ of body mass of protein is consumed in order to cope with demands of a congested fixture period. In order to achieve this amount, an 80 kg player would require approximately 120–160 g of protein per day. Good quality of protein sources such as meat and fish contain around 25 g per 100 g and other sources such as milk, nuts, yoghurt, and beans can contribute to this amount. It has been reported that in elite academy players (U18 s) that there is a skewed distribution of protein intake where more protein is consumed for dinner (~0.6 g·kg^−1^) and lunch (~0.5 g·kg^−1^) in comparison to breakfast (~0.3 g·kg^−1^) [61]. Thus, in terms of the amount of protein consumed over the day, meals or snacks should be divided into 6 × 20–25 g (120–150 g of protein) feedings interspersed by 3 h for stimulating maximal protein synthesis throughout a 24 h period [62].

#### Rehydration after a match – The immediate recovery phase

Intense exercise during a game leads to an increase in metabolic heat production which can raise muscle and rectal temperature to above 39 °C [63]. The main physiological mechanism to lose heat from the body is to evaporate sweat on the skin surface, with losses of 2 L even observed in lower ambient temperatures [64]. As a consequence of this level of fluid loss, a player will become dehydrated. For example, a 75 kg player with sweat losses of >2 L will become dehydrated by >2%. Individual sweat rates can range from 1.1 L to 3.1 L per 90 min [65], outlining the importance of player awareness of their own sweat rate and to rehydrate accordingly post-exercise.

Immediately post-exercise is a period where rehydration strategies should be implemented in order to replace the volume and composition of important fluids lost through sweat. Without adequate rehydration, negative effects on glycogen restoration and protein synthesis rates [66], sprint capacity [67], and subsequent dribbling performance [68] could prevail. It has been reported that at least 150% of the fluid lost during exercise should be consumed to account for a negative fluid balance and urine fluid losses [69]. In practical terms, for every 1 kg of weight lost during exercise would equate to 1.5 L of fluid required post training and this can be monitored through pre-post weighing by support staff.

Time taken to rehydrate is shorter than repletion of muscle glycogen stores (up to 6 h compared to 48–72 h) as long as sufficient fluid and electrolytes are consumed. Although, rehydration may take less time than glycogen re-synthesis, it should be noted that during periods of fixture congestion, especially where teams are playing back to back away fixtures where significant travel is required, it is important to educate players how best to re-hydrate during travel. Moreover, it is not unusual for teams to train 24 h after a match as well as 24 h before a match, placing even greater emphasis on rehydration. Moreover, players should be encouraged to take on adequate fluids during half-time (i.e. 200–300 mL) and throughout the match when opportunities such as a break in play are apparent, to maintain hydration. This is especially important during hot and humid weather conditions.

Sodium is a key electrolyte that should be replaced for optimum fluid restoration. There is a variation amongst players in terms of sodium lost during a game with a reported loss of 10 g of sodium chloride observed during a 90 min soccer session [70]. The consumption of a high sodium drink containing 61 mmol of sodium in volumes equivalent to 150–200% of sweat loss was sufficient to establish a state of hyperhydration 6 h after ingestion [71]. The optimal sodium level during rehydration could be as high as 50–80 mmol·L^−1^ which exceeds the amounts found in a typical sports drink [72]. Water is an electrolyte free drink and is not ideal for rehydration post-exercise as a rapid reduction in plasma sodium concentration could ensure which subsequently increases urine output [73]. Therefore, drinks for rehydration should have high electrolyte content (i.e. 40 or 50 mmol·L^−1^ of sodium chloride) and consist of carbohydrate sources to increase palatability [74] and help with glycogen restoration. In this respect, sports drinks are superior to water for fluid restoration due to their provision of both carbohydrate and electrolytes.

Team sports such as soccer can be associated with a moderate to high post-match alcohol intake to celebrate or commiserate over the game result; especially in the amateur game. Although this practice is slowly diminishing at the elite level, alcohol consumption can negatively affect a player’s ability to recover especially when consumed during periods of fixture congestion [75]. More specifically, alcohol has recently been shown to reduce myofibrillar protein synthesis rates even if coingested with protein, resulting in an impairment of recovery and adaptation from exercise by suppressing skeletal muscle anabolic responses [75]. Moreover, alcohol consumed after a match can also exacerbate dehydration especially when consumed during the recovery period several hours after a match [76]. Thus it is prudent to educate players regarding the negative effects of alcohol on recovery when multiple matches are played within a short period of time.

#### Overnight recovery following match-play

Recovery nutrition towards the end of a day during periods of fixture congestion as well as intensive training is often overlooked by athletes. For instance, protein ingested before sleep has proven to be effectively digested and absorbed, leading to an increase in protein synthesis and improving whole-body protein balance during overnight recovery [49]. Ingesting a pre-sleep protein snack high in casein such as 200 g of cottage cheese or alternatively, a formulated protein supplement containing 40 g of casein protein will likely prove beneficial for increasing the time in a net-positive anabolic state over the course of a day [77]. This is due to its slow release properties over a prolonged sleeping period. The absence of this pre-sleep feed will not improve overnight protein balance; possibly compromising muscle protein synthesis rates over the 24 h period. A summary of the recovery nutrition guidelines have been summarised in Table 3.

### Supplements and recovery

Fundamentally, macro and micro nutrients should come primarily from food sources in the diet; however, players may require a constituent, metabolite, concentrate or extract in isolation that is difficult to source in quantities required from food [78]. While energy consumption from supplements in professional soccer has not been studied, it has been reported that in professional Rugby League players approximately 16% of energy intake came from the use of supplements such as pre-exercise energy drinks, carbohydrate drinks, and recovery drinks [79]. It is important to emphasise that supplements should be consumed to ‘supplement’ a healthy diet and not to replace it. Moreover, elite players should be cautious with supplements and only take batch tested products that have been tested for banned substances. Specific guidelines have yet to be developed with limited research available for the use of some supplements, especially in the context of recovery from elite soccer match-play during periods of fixture congestion. Nevertheless, supplement use during this short recovery phase has become common practice in soccer clubs across a range of ages. Immediately after a match and several hours afterwards, feeding a team with nutritious food can be problematic and therefore certain supplements can be convenient to enhance recovery. A brief review of popular products is provided in this section with reference to their application for recovery.

#### Carbohydrate and protein supplements

Carbohydrate and protein supplements can be both useful and practical for players to enhance recovery during periods of fixture congestion. We have previously discussed both the importance and practical application of carbohydrate and protein supplements under the “recovery nutrition strategies” section.

#### Creatine

During repeated soccer-specific actions phosphocreatine stores diminish significantly as a consequence of adenosine triphosphate regeneration through phosphocreatine hydrolysis in the initial seconds of supra-maximal activity [80]. To increase resting muscle phosphocreatine stores quickly, a creatine loading protocol can be used with the conventional strategy involving 4 × 5 g doses of creatine supplementation per day for 5–7 days proceeded by a maintenance dose of 3–5 g per day [81]. However, a lower daily dose of ~3 g per day for 28 days will result in a similar increase in phosphocreatine stores [81] to the loading protocol. It has been reported that muscle glycogen resynthesis can be enhanced following creatine loading [82]. Practically, creatine can be added to the post-match and post-training recovery drink and it may prove beneficial in optimising refuelling strategies especially during congested fixture schedules.

In agreement with data from the general population [83], empirical observations highlight that sleep deprivation is common on the night(s) prior to sporting competition; especially, if matches require prior international air travel. Interestingly, players who self-reported 7–9 h sleep on the night before testing outperformed their sleep-deprived counterparts (i.e., those reporting 3–5 h sleep) by ~20% in a rugby passing task [84]. Such differences were ameliorated when creatine (50 or 100 mg·kg^−1^) or caffeine (1 or 5 mg·kg^−1^) was provided to sleep-deprived players 90 min before skill testing commenced; a response attributed to the attenuation of sleep-deprivation induced reductions in brain phosphocreatine concentrations and the stimulatory effects of adenosine-receptors, respectively [84].

#### Caffeine

There is some evidence that large amounts of caffeine taken with carbohydrate can enhance glycogen resynthesis post-exercise [85, 86]. Pederson and colleagues [85] found that co-ingestion of carbohydrate and caffeine (4 g·kg^−1^ and 8 mg·kg^−1^, respectively) resulted in greater glycogen resynthesis compared to 4 g·kg^−1^ of body mass of carbohydrate only. Muscle biopsy data showed that although no differences were observed in glycogen resynthesis after 1 h post-exercise (133–37.8 vs. 149–48 mmol·kg^−1^ dry weight; for carbohydrate and caffeine, respectively), after 4 h of recovery the caffeine condition resulted in a 66% higher glycogen accumulation (313–69 vs. 234–50 mmol/kg dry weight; *p* < 0.001). Similarly, Taylor et al. [86] found that co-ingestion of carbohydrate and caffeine (1.2 g·kg^−1^ and 8 mg·kg^−1^, respectively) resulted in an increased time to exhaustion on the Loughborough Intermittent Shuttle Test compared with the carbohydrate only and water condition. Although Taylor et al. [86] did not take any muscle biopsy data to measure glycogen resynthesis, the authors concluded that adding 8 mg·kg^−1^ of caffeine to a post-exercise carbohydrate drink improved subsequent high-intensity interval-running capacity, a finding that may be related to higher rates of post-exercise muscle glycogen resynthesis. Whilst the findings of Pedersen et al. [85] and Taylor et al. [86] may be useful for recovery, as they suggest that adding large doses of caffeine to carbohydrate post-exercise can enhance glycogen resynthesis, this strategy may not always be practical, particularly when matches kick off either late afternoon or evening as this strategy will compromise sleep. Nonetheless, this strategy could be employed for matches that have early kick off times.

#### Antioxidants and polyphenols

When time is limited between games, dietary components that modulate the inflammatory process may prove beneficial in the acute recovery phase. However, it is important to note that any form of antioxidant or anti-inflammatory supplement should be carefully dosed. Soccer-specific adaptations are triggered by the inflammatory and redox reactions occurring after a strenuous exercise stimulus. Therefore, chronically high doses in their provision are likely to be detrimental to the long term training effect [87]. For example, large doses of vitamins C and E have proven to have detrimental effects to cellular adaptation [88, 89]. Strategic use of anti-inflammatory and antioxidant foods/supplements in and around periods of heavy training/game scheduling is the best approach for optimal recovery, rather than chronic daily use.

Antioxidant- and polyphenol-rich foods such as cherry and pomegranate juice have been found to enhance recovery following heavy training [90–94]. For example, 0.682 L a day of tart cherry juice consumption before and after eccentric exercise significantly reduced symptoms of muscle damage [95]. Similarly, Montmorency cherry juice has also been shown to enhance recovery following prolonged, repeat sprint activity in semi-professional male soccer players [91]. In addition, 500 mL of pomegranate juice has been shown to reduce DOMS after strenuous exercise [92, 94]. However, these findings should be interpreted with some caution as participants were fasted and restricted polyphenol based foods beforehand. Theaflavin-enriched black tea extract supplementation in doses of 1760 mg daily for nine days has also been found to enhance recovery, reduce oxidative stress reduce muscle soreness in response to acute anaerobic intervals [96]. Thus, the potential beneficial effects of antioxidants and polyphenols to accelerate recovery are encouraging but more research is warranted using protocols which demonstrate greater ecological validity, especially in relation to soccer specific activity. Nevertheless, in situations where players have back-to-back matches with little time for recovery or in tournament situations where adaptation to training is likely not a key priority, certain antioxidant supplements and polyphenol-rich foods may be beneficial for recovery but chronic use should be avoided.

#### Omega-3 supplementation

Omega-3 is found naturally in oily fish such as salmon, mackerel and sardines, and in a more concentration form as a fish oil supplement. Fish oil supplements contain the long chain polyunsaturated omega-3 fatty acids, eicosapentaenoic acid (EPA) and docosahexaenoic acid (DHA). It should be noted that the research on Omega-3 fatty acid supplements is conflicting as some studies show beneficial effects on reducing inflammation [97] and delayed onset muscle soreness [98–100], whereas, other show no benefit [101, 102]. Phillips and colleagues [97] found that fish oil supplementation reduced exercise-induced inflammation. Similarly, other studies have found that 1.8 g [98], 2.7 g [100], and 3 g [99] of Omega-3 fatty acid supplementation reduced DOMS after exercise. In contrast, other studies have found a reduction in oxidative stress following exercise with fish oil supplementation but no difference in DOMS [102] and further studies have no effect on DOMS [101]. Despite the inconsistencies regarding fish oil supplementation, there does seem to be some evidence for using Omega-3 fatty acid supplementation in doses of 1.8 to 3 g per day to reduce inflammation and muscle soreness after matches, especially during periods of fixture congestion.

### Practical considerations in elite soccer

**Fig. 2:**
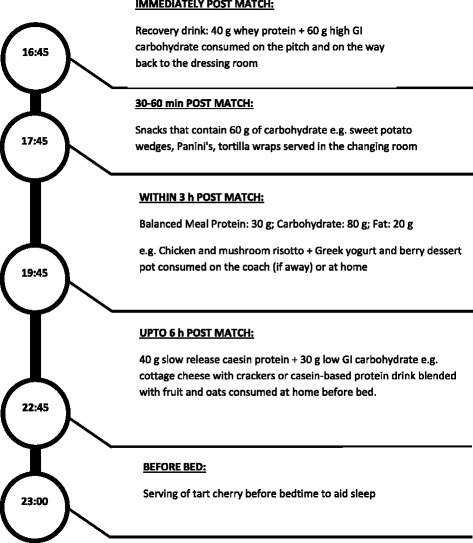
A timeline guide for optimum recovery after match with a kick of time of 15:00 to promote glycogen re-synthesis and repair for an 80 kg player

Fixture scheduling possibly provides the biggest challenge to recovery in elite soccer. It is not unusual for top teams to have 3 games in a 10 day period in 3 different locations (see Fig. 1). The timing of kick offs in these games varies from week to week as a consequence of increased television coverage. For example, a team could play a home match at 15:00 h on a Saturday, travel to Europe to play an away match on Wednesday night at 19:45 h and return to play another away match at 12:45 h on the subsequent Saturday. It is these types of scenarios where recovery strategies take on extra significance. The selection of foods and timing of intake in and around travel are critical factors for optimal recovery. An example of recovery nutrition timeline after a match is shown in Fig. 2. Support staff cannot always rely on external catering thus some foods need to be portable to away games without compromising on quality and in these situations, teams could take their own chef who can work closely with the sport nutritionist to devise suitable menus. Moreover, sleep deprivation will become an issue as a result of late games so timing of recovery nutrition to optimise sleep quality is of significance and this has been reviewed elsewhere [103].

It is easy to formulate a recovery nutrition strategy on paper but implementing it effectively and attaining player adherence in the elite environment can prove a difficult proposition. The role of a sports nutritionist and/or sport science practitioner is to implement these recovery principles by adapting to certain practical restraints (see Table 4 for some practical issues and solutions). The practitioner has to keep in mind that devotion to the right strategies can optimise a player’s physical performance and reduce the risk of fatigue-related injury. This is particularly imperative during a period of congested fixtures where recovery time between matches is limited.

**Table 4:** Practical issues that interfere with post-match recovery nutrition and solutions to counteract these concerns

Practical Issue	Practical Solution
Players within a team who are uneducated and have a detrimental habit of poor quality nutrients during recovery	Educate the team on the importance of recovery nutrition; stressing the beneficial role is had on performance and adaptation. Integrating backroom staff (physiotherapists, coaches, doctor) into this education so that they can re-enforce nutrition policies day to day. Use visuals around the training ground to educate players key messages
Night games limit the time consume recovery nutrients before sleep	Ensure that recovery strategies are implemented every hour until sleep, encouraging more liquid based nutrient sources (e.g. milkshakes, sport supplement drink) to offset any potential gastrointestinal issues associated with the ingestion of solid foods before bed. No caffeinated drinks (e.g. coffee, cola) should be consumed pre-sleep, but a snack containing casein (slow release protein) is important have before bedtime.
Travel to and from away games, sometimes internationally	Recovery snacks need to be carefully chosen so that they are portable and able to travel internationally, without compromising on their quality (e.g. sports bars). Planning is key, ensuring that foods are readily available during transit (e.g. on the team bus or plane). A traveling chef can help enhance quality and taste of meals provided during travel.
Players quickly exiting the stadium to travel home soon after the end of a match (normally in their own car after home games)	Providing a buffet style food selection which provides high-quality sources of mixed carbohydrate and protein snacks. Also, providing a recovery ‘pack’ which contains recovery snacks and/or supplements along with a timing plan for players who have long to travel home.
Players who have been unused sub or not played any minutes	Monitor the minutes played/exercised at higher intensities for all players in the squad. The energy demands and recovery requirements will vary between each individual and should be adjusted accordingly so body composition issues or an energy deficit do not arise. This can be communicated to players using match data
Loss of appetite following high intensity activity	Liquid based nutrient sources such as milkshake and meal replacement shakes should be encouraged to players who don’t have the appetite for food post-match. Again, stress the importance of having recovery nutrition after a game, highlighting the benefits for them as a soccer player (e.g. reduce the risk of injury, improve subsequent performance).
Players choose nutrient-poor foods (i.e. chocolate bar or crisps) because they are more accessible after exercise	Create a culture that promotes good nutrition by using visual displays at the training ground or stadium changing room as well as face to face education. Ensure that a recovery station is set up with high quality food choices (see examples in Table 1) with buffet food selection post game.

At the elite level there is a mix of different cultures and nationalities within a team dressing room but without an empathy and understanding of this environment a player’s match performance and adaptation to exercise can suffer if recovery nutrition is inadequate. To counteract this, a potential strategy could be to introduce a different international theme to recovery snacks/foods at certain points throughout the season for player engagement. This will provide an additional food option during recovery without compromising on the quality of nutrients.

For players, it would also be beneficial to set up a recovery station and buffet style food selection in the changing room after the game which incorporates high-quality sources of carbohydrate and protein recovery snacks. This strategy will ensure that recovery nutrition is readily available after a game before they travel home. It is common practice for some players to quickly exit a game/training almost immediately after exercise so it is important to have this option available. If this option is avoided, a recovery ‘pack’ which contains recovery snacks and/or supplements along with a timing plan could be provided for any players who request; particularly encouraging those who have a long way to travel home and won’t have access to foods.

Support staff may also want to consider an individualised approach to recovery nutrition based on player position. With modern technology such as Global Positioning System (GPS) and data obtained from match analysis such as total distance and high intensity distance covered, recovery strategies could be individualised. For example, players working at higher intensities (typically the full backs, and attacking midfielders) would increase the amount of carbohydrate within the immediate recovery phase. Whereas, the goalkeepers would follow lower carbohydrate diet in order to match the lower energy expenditures.

## Conclusion

The growing match play and training demands of a professional soccer player are putting a greater emphasis on the role of nutritional recovery in regaining performance and reducing the risk of injury. Certain dietary practices should commence immediately after a competitive game or high intensity training session before the opportunity to fully optimise the recuperation process diminishes. Carbohydrate replenishment should take precedence to replace the fuel lost to perform high intensity work with protein consumption playing an important role in muscle repair and rehydration aiding the overall recovery process. Daily strategies incorporating these key nutrients should become common practice on subsequent recovery days between fixtures, especially during congestive weeks. Antioxidants and other nutrients can have a modulating role of the inflammatory process during these busy periods but their use needs be strategic rather than chronic to ensure adaptations to training are not blunted. Current practical issues are ever present in an elite environment and need to be counteracted to achieve success in nutritional approach.
